# Supplementary material for: A Genome-Wide Association Study of Oxypurinol Concentrations in Patients Treated with Allopurinol
Source: J Pers Med. 2024 Jun 18;14(6):649. doi: 10.3390/jpm14060649 (PMC11204675; doi:10.3390/jpm14060649)
Supplement: Supplementary file 1 [file jpm-14-00649-s001.zip › S1 Table.pdf]

Table S1

GWAS results for oxypurinol concentrations (log) in the ALLO-MHI biobank participants.

snp	name	chr	position	region	gene	status	ref	alt	alt_frequency	n	beta	se	t	p
chr1:195308606:A:G	chr1:195308606:A:G	1	195,308,606	intergenic	LINC01031(dist=1942653),LINC01724(dist=736274)	genotyped	A	G	0.4851	439	0.2343	0.0597	65.6523	8.8E-05
rs6673946	chr1:195318450:C:T	1	195,318,450	intergenic	LINC01031(dist=1952497),LINC01724(dist=726430)	imputed	C	T	0.4808	439	0.2372	0.0603	65.3314	8.3E-05
rs12409559	chr1:195322826:C:A	1	195,322,826	intergenic	LINC01031(dist=1956873),LINC01724(dist=722054)	imputed	C	A	0.4737	439	0.2406	0.0605	65.8226	6.9E-05
chr5:98182435:A:G	chr5:98182435:A:G	5	98,182,435	intergenic	LINC01846(dist=21196),RGMB(dist=586197)	imputed	A	G	0.1951	439	0.3320	0.0810	50.5770	4.2E-05
chr5:98186020:T:C	chr5:98186020:T:C	5	98,186,020	intergenic	LINC01846(dist=24781),RGMB(dist=582612)	imputed	T	C	0.1952	439	0.3343	0.0806	51.4668	3.4E-05
chr5:98186031:G:A	chr5:98186031:G:A	5	98,186,031	intergenic	LINC01846(dist=24792),RGMB(dist=582601)	imputed	G	A	0.1972	439	0.3255	0.0800	50.9136	4.7E-05
chr5:98187173:C:A	chr5:98187173:C:A	5	98,187,173	intergenic	LINC01846(dist=25934),RGMB(dist=581459)	imputed	C	A	0.1870	439	0.3350	0.0825	49.1751	4.9E-05
rs10068394	chr5:98189221:A:T	5	98,189,221	intergenic	LINC01846(dist=27982),RGMB(dist=579411)	imputed	A	T	0.1950	439	0.3348	0.0806	51.5502	3.3E-05
chr5:98189936:C:G	chr5:98189936:C:G	5	98,189,936	intergenic	LINC01846(dist=28697),RGMB(dist=578696)	imputed	C	G	0.1950	439	0.3347	0.0806	51.5321	3.3E-05
rs13360217	chr5:98190220:T:C	5	98,190,220	intergenic	LINC01846(dist=28981),RGMB(dist=578412)	imputed	T	C	0.1949	439	0.3347	0.0806	51.5128	3.3E-05
chr5:98191730:A:C	chr5:98191730:A:C	5	98,191,730	intergenic	LINC01846(dist=30491),RGMB(dist=576902)	imputed	A	C	0.1948	439	0.3349	0.0806	51.5034	3.3E-05
chr5:98193996:T:G	chr5:98193996:T:G	5	98,193,996	intergenic	LINC01846(dist=32757),RGMB(dist=574636)	imputed	T	G	0.1950	439	0.3348	0.0806	51.5612	3.3E-05
chr5:98195761:A:G	chr5:98195761:A:G	5	98,195,761	intergenic	LINC01846(dist=34522),RGMB(dist=572871)	imputed	A	G	0.1950	439	0.3348	0.0806	51.5612	3.3E-05
chr5:98207788:T:C	chr5:98207788:T:C	5	98,207,788	intergenic	LINC01846(dist=46549),RGMB(dist=560844)	imputed	T	C	0.1955	439	0.3333	0.0804	51.6271	3.3E-05
chr5:98208195:T:A	chr5:98208195:T:A	5	98,208,195	intergenic	LINC01846(dist=46956),RGMB(dist=560437)	imputed	T	A	0.1987	439	0.3164	0.0795	50.1203	6.8E-05
rs3923901	chr5:98210349:T:C	5	98,210,349	intergenic	LINC01846(dist=49110),RGMB(dist=558283)	imputed	T	C	0.1955	439	0.3333	0.0803	51.6304	3.4E-05
chr5:98218328:T:C	chr5:98218328:T:C	5	98,218,328	intergenic	LINC01846(dist=57089),RGMB(dist=550304)	imputed	T	C	0.1955	439	0.3331	0.0803	51.6283	3.4E-05
chr5:98219586:A:C	chr5:98219586:A:C	5	98,219,586	intergenic	LINC01846(dist=58347),RGMB(dist=549046)	genotyped	A	C	0.1970	439	0.3308	0.0798	52.0126	3.4E-05
chr5:98221623:C:A	chr5:98221623:C:A	5	98,221,623	intergenic	LINC01846(dist=60384),RGMB(dist=547009)	imputed	C	A	0.1955	439	0.3331	0.0803	51.6130	3.4E-05
chr5:98222613:A:C	chr5:98222613:A:C	5	98,222,613	intergenic	LINC01846(dist=61374),RGMB(dist=546019)	imputed	A	C	0.1955	439	0.3331	0.0803	51.6130	3.4E-05
chr5:98226299:GA:G	chr5:98226299:GA:G	5	98,226,299	intergenic	LINC01846(dist=65060),RGMB(dist=542333)	imputed	GA	G	0.1953	439	0.3330	0.0805	51.3455	3.5E-05
chr5:98230523:G:A	chr5:98230523:G:A	5	98,230,523	intergenic	LINC01846(dist=69284),RGMB(dist=538109)	imputed	G	A	0.1952	439	0.3332	0.0805	51.4502	3.5E-05
chr5:98231356:T:C	chr5:98231356:T:C	5	98,231,356	intergenic	LINC01846(dist=70117),RGMB(dist=537276)	imputed	T	C	0.1951	439	0.3334	0.0805	51.4658	3.4E-05
chr5:98231514:T:C	chr5:98231514:T:C	5	98,231,514	intergenic	LINC01846(dist=70275),RGMB(dist=537118)	imputed	T	C	0.1951	439	0.3324	0.0805	51.2792	3.6E-05
chr5:98231576:A:C	chr5:98231576:A:C	5	98,231,576	intergenic	LINC01846(dist=70337),RGMB(dist=537056)	imputed	A	C	0.1951	439	0.3336	0.0806	51.3920	3.5E-05
chr5:98232413:A:C	chr5:98232413:A:C	5	98,232,413	intergenic	LINC01846(dist=71174),RGMB(dist=536219)	imputed	A	C	0.1960	439	0.3305	0.0804	51.0759	4.0E-05
chr5:98232517:T:C	chr5:98232517:T:C	5	98,232,517	intergenic	LINC01846(dist=71278),RGMB(dist=536115)	imputed	T	C	0.1963	439	0.3301	0.0803	51.1347	4.0E-05
chr5:98233071:A:G	chr5:98233071:A:G	5	98,233,071	intergenic	LINC01846(dist=71832),RGMB(dist=535561)	imputed	A	G	0.1952	439	0.3332	0.0805	51.4415	3.5E-05
rs36159195	chr5:98234162:A:T	5	98,234,162	intergenic	LINC01846(dist=72923),RGMB(dist=534470)	imputed	A	T	0.2050	439	0.3257	0.0788	52.4976	3.5E-05
rs139485214	chr5:98234650:T:G	5	98,234,650	intergenic	LINC01846(dist=73411),RGMB(dist=533982)	imputed	T	G	0.2062	439	0.3227	0.0786	52.1980	4.1E-05
chr5:98235261:C:T	chr5:98235261:C:T	5	98,235,261	intergenic	LINC01846(dist=74022),RGMB(dist=533371)	imputed	C	T	0.2039	439	0.3283	0.0789	52.7737	3.2E-05
rs140554133	chr5:98236515:C:T	5	98,236,515	intergenic	LINC01846(dist=75276),RGMB(dist=532117)	imputed	C	T	0.2039	439	0.3282	0.0789	52.7686	3.2E-05
chr5:98237144:C:T	chr5:98237144:C:T	5	98,237,144	intergenic	LINC01846(dist=75905),RGMB(dist=531488)	imputed	C	T	0.2082	439	0.3176	0.0786	51.4538	5.3E-05
rs140679700	chr5:98237303:C:T	5	98,237,303	intergenic	LINC01846(dist=76064),RGMB(dist=531329)	imputed	C	T	0.2062	439	0.3225	0.0786	52.1593	4.1E-05
**chr5:98238797:G:A**	**chr5:98238797:G:A**	**5**	**98,238,797**	**intergenic**	**LINC01846(dist=77558),RGMB(dist=529835)**	**imputed**	**G**	**A**	**0.2038**	**439**	**0.3317**	**0.0794**	**52.6326**	**2.9E-05**
chr5:98239348:C:T	chr5:98239348:C:T	5	98,239,348	intergenic	LINC01846(dist=78109),RGMB(dist=529284)	imputed	C	T	0.2055	439	0.3249	0.0791	51.9032	4.0E-05
rs375151094	chr5:98239595:A:G	5	98,239,595	intergenic	LINC01846(dist=78356),RGMB(dist=529037)	imputed	A	G	0.2076	439	0.3126	0.0785	50.7718	6.8E-05
rs369789810	chr5:98239733:C:T	5	98,239,733	intergenic	LINC01846(dist=78494),RGMB(dist=528899)	imputed	C	T	0.2032	439	0.3306	0.0794	52.4915	3.1E-05
chr5:98239906:G:A	chr5:98239906:G:A	5	98,239,906	intergenic	LINC01846(dist=78667),RGMB(dist=528726)	imputed	G	A	0.2065	439	0.3150	0.0786	51.0426	6.1E-05
chr5:98240302:A:G	chr5:98240302:A:G	5	98,240,302	intergenic	LINC01846(dist=79063),RGMB(dist=528330)	imputed	A	G	0.2076	439	0.3126	0.0785	50.7817	6.8E-05
chr5:98240496:G:C	chr5:98240496:G:C	5	98,240,496	intergenic	LINC01846(dist=79257),RGMB(dist=528136)	imputed	G	C	0.2032	439	0.3306	0.0794	52.4827	3.1E-05
chr5:98240901:T:A	chr5:98240901:T:A	5	98,240,901	intergenic	LINC01846(dist=79662),RGMB(dist=527731)	imputed	T	A	0.2076	439	0.3122	0.0784	50.7594	6.9E-05
rs142086196	chr5:98241782:C:T	5	98,241,782	intergenic	LINC01846(dist=80543),RGMB(dist=526850)	imputed	C	T	0.2032	439	0.3306	0.0794	52.4930	3.1E-05
chr5:98244457:G:T	chr5:98244457:G:T	5	98,244,457	intergenic	LINC01846(dist=83218),RGMB(dist=524175)	imputed	G	T	0.2032	439	0.3306	0.0794	52.4713	3.1E-05
rs36172267	chr5:98246772:G:A	5	98,246,772	intergenic	LINC01846(dist=85533),RGMB(dist=521860)	imputed	G	A	0.2032	439	0.3306	0.0794	52.4872	3.1E-05
chr5:98247104:A:G	chr5:98247104:A:G	5	98,247,104	intergenic	LINC01846(dist=85865),RGMB(dist=521528)	imputed	A	G	0.2055	439	0.3249	0.0791	51.9111	4.0E-05
rs28970359	chr5:98249364:C:T	5	98,249,364	intergenic	LINC01846(dist=88125),RGMB(dist=519268)	imputed	C	T	0.2016	439	0.3275	0.0801	51.0746	4.3E-05
chr5:98249758:C:T	chr5:98249758:C:T	5	98,249,758	intergenic	LINC01846(dist=88519),RGMB(dist=518874)	imputed	C	T	0.2016	439	0.3276	0.0801	51.0686	4.3E-05
chr5:98251313:T:C	chr5:98251313:T:C	5	98,251,313	intergenic	LINC01846(dist=90074),RGMB(dist=517319)	imputed	T	C	0.2044	439	0.3277	0.0792	52.2212	3.5E-05
chr5:98254474:T:C	chr5:98254474:T:C	5	98,254,474	intergenic	LINC01846(dist=93235),RGMB(dist=514158)	imputed	T	C	0.2044	439	0.3276	0.0792	52.2154	3.5E-05
chr5:98254894:G:T	chr5:98254894:G:T	5	98,254,894	intergenic	LINC01846(dist=93655),RGMB(dist=513738)	imputed	G	T	0.2015	439	0.3278	0.0801	51.0691	4.3E-05
chr5:98255322:T:G	chr5:98255322:T:G	5	98,255,322	intergenic	LINC01846(dist=94083),RGMB(dist=513310)	imputed	T	G	0.2044	439	0.3274	0.0792	52.1681	3.6E-05
chr5:98256950:G:T	chr5:98256950:G:T	5	98,256,950	intergenic	LINC01846(dist=95711),RGMB(dist=511682)	imputed	G	T	0.2008	439	0.3294	0.0806	50.7534	4.3E-05

snp	name	chr	position	region	gene	status	ref	alt	alt_frequency	n	beta	se	t	p
chr5:98257307:A:G	chr5:98257307:A:G	5	98,257,307	intergenic	LINC01846(dist=96068),RGMB(dist=511325)	imputed	A	G	0.2026	439	0.3321	0.0798	52.1351	3.2E-05
chr5:98259083:C:T	chr5:98259083:C:T	5	98,259,083	intergenic	LINC01846(dist=97844),RGMB(dist=509549)	imputed	C	T	0.2036	439	0.3293	0.0797	51.8412	3.6E-05
rs7701695	chr5:98261085:G:C	5	98,261,085	intergenic	LINC01846(dist=99846),RGMB(dist=507547)	imputed	G	C	0.2002	439	0.3234	0.0814	48.7924	7.1E-05
chr13:38086490:C:T	chr13:38086490:C:T	13	38,086,490	ncRNA_intronic	LINC00571	imputed	C	T	0.2009	439	0.2981	0.0752	52.7221	7.4E-05
rs17057180	chr13:38094190:C:T	13	38,094,190	ncRNA_intronic	LINC00571	imputed	C	T	0.1395	439	0.3433	0.0878	44.5155	9.3E-05
chr13:38095957:G:T	chr13:38095957:G:T	13	38,095,957	ncRNA_intronic	LINC00571	imputed	G	T	0.1395	439	0.3431	0.0878	44.4944	9.3E-05
chr13:38096500:A:G	chr13:38096500:A:G	13	38,096,500	ncRNA_intronic	LINC00571	imputed	A	G	0.1396	439	0.3431	0.0878	44.5003	9.3E-05
chr13:38098236:A:G	chr13:38098236:A:G	13	38,098,236	ncRNA_intronic	LINC00571	imputed	A	G	0.2018	439	0.2980	0.0751	52.8913	7.2E-05
chr13:38098318:A:G	chr13:38098318:A:G	13	38,098,318	ncRNA_intronic	LINC00571	imputed	A	G	0.2018	439	0.2979	0.0751	52.8838	7.2E-05
chr13:38098483:A:G	chr13:38098483:A:G	13	38,098,483	ncRNA_intronic	LINC00571	imputed	A	G	0.2018	439	0.2980	0.0751	52.8885	7.2E-05
rs7334407	chr13:38109210:T:C	13	38,109,210	ncRNA_intronic	LINC00571	imputed	T	C	0.2011	439	0.2954	0.0753	52.0764	8.8E-05
rs9603323	chr13:38112298:G:A	13	38,112,298	ncRNA_intronic	LINC00571	imputed	G	A	0.1393	439	0.3437	0.0879	44.4756	9.2E-05
chr13:38112460:G:A	chr13:38112460:G:A	13	38,112,460	ncRNA_intronic	LINC00571	imputed	G	A	0.2098	439	0.2876	0.0736	53.0612	9.4E-05
chr13:38117402:G:A	chr13:38117402:G:A	13	38,117,402	ncRNA_intronic	LINC00571	imputed	G	A	0.2005	439	0.2965	0.0755	52.0681	8.5E-05
chr13:38167941:T:C	chr13:38167941:T:C	13	38,167,941	intergenic	LINC00571(dist=24709),UFM1(dist=181910)	imputed	T	C	0.2010	439	0.3010	0.0773	50.3757	9.9E-05

Abbreviations: chr, chromosome; dist, distance; ref, reference allele; alt, alternative allele; se, standard error.

Note: all variant results with statistical significance $p<10^{-4}$ are displayed. Only the variant with the highest significance is in bold.
