# Supplementary material for: A Genome-Wide Association Study of Oxypurinol Concentrations in Patients Treated with Allopurinol
Source: J Pers Med. 2024 Jun 18;14(6):649. doi: 10.3390/jpm14060649 (PMC11204675; doi:10.3390/jpm14060649)
Supplement: Supplementary file 1 [file jpm-14-00649-s001.zip › S2 Table.pdf]

Table S2

GWAS results for allopurinol concentrations (log) in the ALLO-MHI biobank participants.

snp	name	chr	position	region	gene	status	ref	alt	alt_frequency	n	beta	se	t	p
rs73179928	chr3:121561196:A:G	3	121,561,196	intergenic	POLQ(dist=15208),ARGFX(dist=6753)	genotyped	A	G	0.0524	439	-0.5914	0.1322	-33.8646	7.6E-06
rs73179930	chr3:121562551:A:T	3	121,562,551	intergenic	POLQ(dist=16563),ARGFX(dist=5398)	imputed	A	T	0.0522	439	-0.5947	0.1327	-33.7902	7.4E-06
chr3:121564544:G:C	chr3:121564544:G:C	3	121,564,544	intergenic	POLQ(dist=18556),ARGFX(dist=3405)	imputed	G	C	0.0522	439	-0.5947	0.1327	-33.7822	7.4E-06
chr3:121566934:G:C	chr3:121566934:G:C	3	121,566,934	intergenic	POLQ(dist=20946),ARGFX(dist=1015)	imputed	G	C	0.0507	439	-0.6112	0.1368	-32.6729	7.9E-06
rs61559512	chr4:152207259:T:C	4	152,207,259	intergenic	LINC02273(dist=102543),FBXW7(dist=113285)	imputed	T	C	0.2631	439	-0.3033	0.0681	-65.4171	8.4E-06
rs7743540	chr6:49561207:C:T	6	49,561,207	intergenic	C6orf141(dist=9112),RHAG(dist=43970)	imputed	C	T	0.5610	439	-0.2880	0.0595	-81.3800	1.3E-06
**chr6:49561859:T:C**	**chr6:49561859:T:C**	**6**	**49,561,859**	**intergenic**	**C6orf141(dist=9764),RHAG(dist=43318)**	**imputed**	**T**	**C**	**0.5609**	**439**	**-0.2884**	**0.0595**	**-81.4402**	**1.3E-06**
chr6:49565759:A:T	chr6:49565759:A:T	6	49,565,759	intergenic	C6orf141(dist=13664),RHAG(dist=39418)	imputed	A	T	0.5603	439	-0.2830	0.0593	-80.5745	1.8E-06
rs9463502	chr6:49572703:A:T	6	49,572,703	intergenic	C6orf141(dist=20608),RHAG(dist=32474)	imputed	A	T	0.5604	439	-0.2833	0.0593	-80.6650	1.7E-06
chr6:49572868:G:A	chr6:49572868:G:A	6	49,572,868	intergenic	C6orf141(dist=20773),RHAG(dist=32309)	imputed	G	A	0.5605	439	-0.2829	0.0593	-80.5816	1.8E-06
rs7775756	chr6:49572992:C:T	6	49,572,992	intergenic	C6orf141(dist=20897),RHAG(dist=32185)	imputed	C	T	0.5605	439	-0.2829	0.0593	-80.5766	1.8E-06
chr6:49573166:A:T	chr6:49573166:A:T	6	49,573,166	intergenic	C6orf141(dist=21071),RHAG(dist=32011)	imputed	A	T	0.5605	439	-0.2829	0.0593	-80.5860	1.8E-06
rs6458702	chr6:49573373:G:A	6	49,573,373	intergenic	C6orf141(dist=21278),RHAG(dist=31804)	genotyped	G	A	0.5638	439	-0.2802	0.0590	-80.5015	2.0E-06
chr6:49579644:G:T	chr6:49579644:G:T	6	49,579,644	intergenic	C6orf141(dist=27549),RHAG(dist=25533)	imputed	G	T	0.5964	439	-0.2987	0.0616	-78.6002	1.3E-06
chr7:157402463:A:G	chr7:157402463:A:G	7	157,402,463	intronic	DNAJB6	imputed	A	G	0.1472	439	0.4238	0.0893	53.1429	2.1E-06
chr7:157402521:A:G	chr7:157402521:A:G	7	157,402,521	intronic	DNAJB6	imputed	A	G	0.1458	439	0.4261	0.0896	53.1290	2.0E-06
rs7782695	chr7:157402526:T:C	7	157,402,526	intronic	DNAJB6	imputed	T	C	0.1458	439	0.4260	0.0896	53.1235	2.0E-06
chr7:157402616:T:C	chr7:157402616:T:C	7	157,402,616	intronic	DNAJB6	imputed	T	C	0.1458	439	0.4260	0.0895	53.1390	2.0E-06
rs4716718	chr7:157402628:G:T	7	157,402,628	intronic	DNAJB6	imputed	G	T	0.1458	439	0.4260	0.0895	53.1390	2.0E-06
chr8:8741890:G:T	chr8:8741890:G:T	8	8,741,890	intergenic	CLDN23(dist=37794),MFHAS1(dist=41465)	imputed	G	T	0.0951	439	0.4676	0.0998	46.9544	2.8E-06
rs118115974	chr9:121609349:G:A	9	121,609,349	intronic	DAB2IP	genotyped	G	A	0.0501	439	-0.5925	0.1338	-33.1073	9.5E-06
chr12:42669224:C:A	chr12:42669224:C:A	12	42,669,224	ncRNA_intronic	LINC02451	imputed	C	A	0.7922	439	-0.3467	0.0756	-60.7272	4.5E-06
chr12:42669734:T:A	chr12:42669734:T:A	12	42,669,734	ncRNA_intronic	LINC02451	imputed	T	A	0.8205	439	-0.3720	0.0774	-62.1317	1.5E-06
chr12:42673164:T:C	chr12:42673164:T:C	12	42,673,164	ncRNA_intronic	LINC02451	imputed	T	C	0.8197	439	-0.3732	0.0772	-62.6560	1.3E-06
rs73129689	chr12:42673598:G:A	12	42,673,598	ncRNA_intronic	LINC02451	imputed	G	A	0.1086	439	0.4369	0.0946	48.7952	3.9E-06
chr12:42677591:G:T	chr12:42677591:G:T	12	42,677,591	ncRNA_intronic	LINC02451	genotyped	G	T	0.8269	439	-0.3623	0.0772	-60.7706	2.7E-06
rs4581703	chr16:25961704:A:C	16	25,961,704	intronic	HS3ST4	imputed	A	C	0.1794	439	0.3517	0.0778	58.0291	6.3E-06
chr16:25961806:CA:C	chr16:25961806:CA:C	16	25,961,806	intronic	HS3ST4	imputed	CA	C	0.1806	439	0.3499	0.0779	57.7135	7.0E-06
rs4578653	chr16:25961890:A:G	16	25,961,890	intronic	HS3ST4	imputed	A	G	0.1805	439	0.3446	0.0771	57.9784	7.8E-06
rs3923425	chr16:25962716:C:A	16	25,962,716	intronic	HS3ST4	genotyped	C	A	0.1800	439	0.3510	0.0775	58.3696	6.0E-06
chr16:25963155:C:T	chr16:25963155:C:T	16	25,963,155	intronic	HS3ST4	imputed	C	T	0.1793	439	0.3522	0.0778	58.1614	6.0E-06
rs4787791	chr16:25964047:T:C	16	25,964,047	intronic	HS3ST4	imputed	T	C	0.1793	439	0.3523	0.0778	58.1549	6.0E-06
chr18:37076582:G:A	chr18:37076582:G:A	18	37,076,582	intronic	KIAA1328	imputed	G	A	0.3365	439	0.3063	0.0660	70.2884	3.5E-06

Abbreviations: chr, chromosome; dist, distance; ref, reference allele; alt, alternative allele; se, standard error.

Note: all variant results with statistical significance $p<10^{-4}$ are displayed. Only the variant with the highest significance is in bold.
