# Supplementary material for: A Genome-Wide Association Study of Oxypurinol Concentrations in Patients Treated with Allopurinol
Source: J Pers Med. 2024 Jun 18;14(6):649. doi: 10.3390/jpm14060649 (PMC11204675; doi:10.3390/jpm14060649)
Supplement: Supplementary file 1 [file jpm-14-00649-s001.zip › S3 Table.pdf]

Table S3

GWAS results for allopurinol-riboside concentrations (log) in the ALLO-MHI biobank participants.

snp	name	chr	position	region	gene	status	ref	alt	alt_frequency	n	beta	se	t	p
rs6972273	chr7:8404132:C:T	7	8,404,132	intergenic	LOC100505938(dist=60851),NXPH1(dist=29477)	imputed	C	T	0.6240	439	-0.3338	0.0671	-74.1051	6.6E-07
chr7:8404703:C:T	chr7:8404703:C:T	7	8,404,703	intergenic	LOC100505938(dist=61422),NXPH1(dist=28906)	imputed	C	T	0.6229	439	-0.3308	0.0672	-73.2637	8.5E-07
**chr7:8405423:G:A**	**chr7:8405423:G:A**	**7**	**8,405,423**	**intergenic**	**LOC100505938(dist=62142),NXPH1(dist=28186)**	**imputed**	**G**	**A**	**0.6240**	**439**	**-0.3339**	**0.0671**	**-74.1147**	**6.5E-07**
chr7:8406761:T:A	chr7:8406761:T:A	7	8,406,761	intergenic	LOC100505938(dist=63480),NXPH1(dist=26848)	imputed	T	A	0.6252	439	-0.3297	0.0674	-72.5820	1.0E-06
chr7:8408325:C:T	chr7:8408325:C:T	7	8,408,325	intergenic	LOC100505938(dist=65044),NXPH1(dist=25284)	imputed	C	T	0.6228	439	-0.3332	0.0672	-73.7297	7.2E-07

Abbreviations: chr, chromosome; dist, distance; ref, reference allele; alt, alternative allele; se, standard error.

Note: all variant results with statistical significance $p<10^{-4}$ are displayed. Only the variant with the highest significance is in bold.
