# Supplementary material for: A Genome-Wide Association Study of Oxypurinol Concentrations in Patients Treated with Allopurinol
Source: J Pers Med. 2024 Jun 18;14(6):649. doi: 10.3390/jpm14060649 (PMC11204675; doi:10.3390/jpm14060649)
Supplement: Supplementary file 1 [file jpm-14-00649-s001.zip › S4 Table.pdf]

Table S4

GWAS results for daily allopurinol dose (log) in the ALLO-MHI biobank participants.

snp	name	chr	position	region	gene	status	ref	alt	alt_frequency	n	beta	se	t	p
chr6:32663671:A:G	chr6:32663671:A:G	6	32,663,671	intronic	HLA-DQB1	imputed	A	G	0.3235	439	0.3066	0.0625	78.4237	9.4E-07
rs1570303	chr9:111638718:G:A	9	111,638,718	intronic	DNAJC25,DNAJC25-GNG10	genotyped	G	A	0.1424	439	-0.3947	0.0804	-61.0109	9.2E-07
rs11159972	chr14:26714519:A:G	14	26,714,519	ncRNA_intronic	LINC02588	imputed	A	G	0.3680	439	0.3134	0.0607	85.0418	2.4E-07
rs1950413	chr14:26715593:A:G	14	26,715,593	ncRNA_intronic	LINC02588	imputed	A	G	0.3697	439	0.3057	0.0604	83.8353	4.1E-07
rs12588122	chr14:26716268:C:T	14	26,716,268	ncRNA_intronic	LINC02588	imputed	C	T	0.3561	439	0.3118	0.0601	86.2507	2.2E-07
chr14:26717773:G:T	chr14:26717773:G:T	14	26,717,773	ncRNA_intronic	LINC02588	genotyped	G	T	0.4193	439	0.2940	0.0589	84.6432	6.1E-07
rs12590159	chr14:26719151:C:T	14	26,719,151	ncRNA_intronic	LINC02588	imputed	C	T	0.3545	439	0.3184	0.0605	87.0366	1.4E-07
chr14:26720060:G:A	chr14:26720060:G:A	14	26,720,060	ncRNA_intronic	LINC02588	imputed	G	A	0.4179	439	0.2915	0.0594	82.6157	9.2E-07
rs61453612	chr14:26720447:C:T	14	26,720,447	ncRNA_intronic	LINC02588	imputed	C	T	0.4145	439	0.2994	0.0594	84.8269	4.7E-07
rs58397259	chr14:26720477:A:C	14	26,720,477	ncRNA_intronic	LINC02588	imputed	A	C	0.3695	439	0.3056	0.0604	83.6645	4.3E-07
rs8007646	chr14:26721866:T:C	14	26,721,866	ncRNA_intronic	LINC02588	imputed	T	C	0.4385	439	0.3147	0.0593	89.5471	1.1E-07
chr14:26722162:A:G	chr14:26722162:A:G	14	26,722,162	ncRNA_intronic	LINC02588	imputed	A	G	0.4157	439	0.2950	0.0595	83.2834	7.2E-07
chr14:26722613:G:T	chr14:26722613:G:T	14	26,722,613	ncRNA_intronic	LINC02588	imputed	G	T	0.4384	439	0.3154	0.0594	89.3979	1.1E-07
chr14:26722750:T:G	chr14:26722750:T:G	14	26,722,750	ncRNA_intronic	LINC02588	imputed	T	G	0.4384	439	0.3156	0.0594	89.4501	1.1E-07
rs12589798	chr14:26724183:G:A	14	26,724,183	ncRNA_intronic	LINC02588	imputed	G	A	0.4155	439	0.2976	0.0596	83.7017	6.0E-07
chr14:26724507:C:T	chr14:26724507:C:T	14	26,724,507	ncRNA_intronic	LINC02588	imputed	C	T	0.4156	439	0.2975	0.0596	83.7089	6.0E-07
rs72673139	chr14:26729719:G:A	14	26,729,719	ncRNA_intronic	LINC02588	imputed	G	A	0.4167	439	0.3014	0.0595	85.2149	4.0E-07
**rs72673145**	**chr14:26734822:G:A**	**14**	**26,734,822**	**ncRNA_intronic**	**LINC02588**	**imputed**	**G**	**A**	**0.3700**	**439**	**0.3217**	**0.0605**	**87.8486**	**1.1E-07**
chr14:26735981:G:A	chr14:26735981:G:A	14	26,735,981	ncRNA_intronic	LINC02588	imputed	G	A	0.3554	439	0.3166	0.0609	85.3039	2.0E-07
rs4141700	chr14:26736497:T:C	14	26,736,497	ncRNA_intronic	LINC02588	imputed	T	C	0.3828	439	0.3245	0.0612	86.7646	1.1E-07

Abbreviations: chr, chromosome; dist, distance; ref, reference allele; alt, alternative allele; se, standard error.

Note: all variant results with statistical significance $p<10^{-4}$ are displayed. Only the variant with the highest significance is in bold.
