# Supplementary material for: A Genome-Wide Association Study of Oxypurinol Concentrations in Patients Treated with Allopurinol
Source: J Pers Med. 2024 Jun 18;14(6):649. doi: 10.3390/jpm14060649 (PMC11204675; doi:10.3390/jpm14060649)
Supplement: Supplementary file 1 [file jpm-14-00649-s001.zip › S5 Table.pdf]

Table S5

GWAS results of genetic lookups for oxypurinol concentrations (log) using a linear regression with ALLO-MHI biobank participants.

CHR	POS	rsID	Allele 1	Allele 2	Allele 2 frequency	N	BETA	SE	Tstat	p-value*	SNP	Gene	imputed
1	145711327	chr1:145711327:T:G	T	G	0.46355	439	-0.00952	0.0613	-2.53406	0.87655	rs1471633	PDZK1	no
1	155179017	chr1:155179017:C:T	C	T	0.3747197	439	-0.03302	0.06222	-8.53012	0.59559	rs11264341	TRIM46	no
1	240558252	chr1:240558252:C:T	C	T	0.31497	439	-0.03994	0.06909	-8.36545	0.56327	rs1934341	GREM2	yes
2	27508073	chr2:27508073:T:C	T	C	0.5072	439	-0.01737	0.05966	-4.8808	0.77091	rs1260326	GKCR	no
2	27518370	chr2:27518370:T:C	T	C	0.5205	439	-0.0043	0.05998	-1.19441	0.94289	rs780094	GKCR	no
2	120548864	chr2:120548864:G:A	G	A	0.4285095	439	-0.05624	0.06185	-14.70067	0.3632	rs17050272	INHBB	no
3	53066198	chr3:53066198:G:T	G	T	0.5671937	439	0.00181	0.06095	0.48734	0.9763	rs6770152	SFMBT1	no
4	9914117	chr4:9914117:C:T	C	T	0.38497	439	-0.00939	0.06088	-2.53413	0.8774	rs11722228	SLC2A9	no
4	9942428	chr4:9942428:A:G	A	G	0.1728795	439	0.06937	0.07991	10.86288	0.38534	rs12498742	SLC2A9	yes
4	9961141	chr4:9961141:T:C	T	C	0.15831	439	0.06061	0.08263	8.87752	0.46322	rs11942223	SLC2A9	no
4	9993558	chr4:9993558:G:C	G	C	0.82485	439	-0.04168	0.08221	-6.16744	0.61215	rs3775948	SLC2A9	no
5	73135655	chr5:73135655:G:C	G	C	0.2831971	439	0.10135	0.07351	18.75512	0.16799	rs17632159	TMEM171	yes
6	7101851	chr6:7101851:T:C	T	C	0.6996829	439	0.005	0.06501	1.18356	0.93867	rs675209	RREB1	no
6	25821388	chr6:25821388:T:G	T	G	0.5542007	439	0.02723	0.06123	7.26298	0.65655	rs1165151	SLC17A1	yes
6	25823216	chr6:25823216:A:T	A	T	0.55199	439	0.03113	0.06147	8.23887	0.61254	rs1183201	SLC17A	no
6	43836834	chr6:43836834:T:G	T	G	0.7357676	439	0.05344	0.07067	10.6994	0.44956	rs729761	VEGFA	no
7	73442719	chr7:73442719:A:G	A	G	0.1446469	439	-0.03297	0.08236	-4.86075	0.68891	rs1178977	BAZ1B	no
7	151708919	chr7:151708919:C:T	C	T	0.2915182	439	-0.08216	0.06449	-19.75456	0.20268	rs10480300	PRKAG2	no
8	23919493	chr8:23919493:A:G	A	G	0.429385	439	0.00546	0.06435	1.31875	0.93237	rs17786744	STC1	no
8	75566533	chr8:75566533:C:T	C	T	0.4544419	439	-0.02062	0.0569	-6.36815	0.71709	rs2941484	HNF4G	no
10	50886333	chr10:50886333:G:A	G	A	0.1893385	439	0.00695	0.07597	1.2052	0.92705	rs10821905	A1CF	yes
10	59709780	chr10:59709780:T:C	T	C	0.7744875	439	-0.07365	0.0729	-13.86041	0.31232	rs1171614	SLC16A9	no
11	64563990	chr11:64563990:A:G	A	G	0.46583	439	0.02133	0.06047	5.83269	0.72433	rs17300741	SLC22A11	no
11	64566642	chr11:64566642:C:T	C	T	0.4658314	439	0.02073	0.06051	5.66228	0.73187	rs2078267	SLC22A11	no
11	64591337	chr11:64591337:G:A	G	A	0.68827	439	0.08235	0.06652	18.61195	0.2157	rs3825018	SLC22A12	yes
11	64710591	chr11:64710591:G:A	G	A	0.851905	439	0.06345	0.08395	9.00391	0.44973	rs478607	NRXN2	no
11	64778919	chr11:64778919:C:T	C	T	0.87015	439	0.02286	0.09063	2.78352	0.80084	rs606458	SF1	no
12	51857488	chr12:51857488:G:T	G	T	0.3029255	439	-0.07578	0.0634	-18.8532	0.23196	rs7976059	ACVR1B	no
12	57450266	chr12:57450266:C:T	C	T	0.1890839	439	-0.09445	0.07926	-15.03327	0.23342	rs3741414	INHBC	no
12	111569952	chr12:111569952:C:T	C	T	0.5011256	439	-0.03815	0.05998	-10.6042	0.52475	rs653178	ATXN2	no
12	122141445	chr12:122141445:A:G	A	G	0.5045558	439	0.03042	0.0603	8.36794	0.61387	rs7953704	B3GNT4	no
15	75866642	chr15:75866642:G:A	G	A	0.3915628	439	-0.02431	0.06322	-6.08272	0.70059	rs1394125	UBE2Q2	no
15	98727906	chr15:98727906:A:G	A	G	0.6115146	439	-0.02554	0.06165	-6.72125	0.67863	rs6598541	IGF1R	no
16	69529987	chr16:69529987:C:T	C	T	0.8565054	439	-0.0115	0.08302	-1.66833	0.88985	rs7193778	NFAT5	no
16	79701090	chr16:79701090:G:A	G	A	0.3162267	439	-0.03415	0.06795	-7.39539	0.6153	rs7188445	MAF	yes
17	55287427	chr17:55287427:C:A	C	A	0.5710036	439	0.02241	0.06076	6.07003	0.71227	rs7224610	HLF	no

*Adjusted for age, sex, weight, daily allopurinol dose, and PC1-10 (n=439 samples).

Gene information was retrieved with Annovar (version 2020-06-07). The rsID were retrieved with Ensembl REST API.

Abbreviations: CHR, chromosome; POS, position SE, standard error.
