# Supplementary material for: A Genome-Wide Association Study of Oxypurinol Concentrations in Patients Treated with Allopurinol
Source: J Pers Med. 2024 Jun 18;14(6):649. doi: 10.3390/jpm14060649 (PMC11204675; doi:10.3390/jpm14060649)
Supplement: Supplementary file 1 [file jpm-14-00649-s001.zip › S6 Table.pdf]

Table S6

GWAS results of genetic lookups for allopurinol concentrations (log) using a linear regression with ALLO-MHI biobank participants.

CHR	POS	rsID	Allele 1	Allele 2	Allele 2 frequency	N	BETA	SE	Tstat	p-value*	SNP	Gene	imputed
1	145711327	chr1:145711327:T:G	T	G	0.4636	439	-0.03485	0.058925	-10.0357	0.554287	rs1471633	PDZK1	no
1	240558252	chr1:240558252:C:T	C	T	0.315	439	-0.07929	0.066124	-18.135	0.230464	rs1934341	GREM2	yes
1	155179017	chr1:155179017:C:T	C	T	0.3747	439	0.0103	0.0599	2.8621	0.8638	rs11264341	TRIM46	no
2	27508073	chr2:27508073:T:C	T	C	0.5072	439	0.060056	0.057533	18.1433	0.296558	rs1260326	GKCR	no
2	27518370	chr2:27518370:T:C	T	C	0.5205	439	0.043054	0.057828	12.87465	0.456566	rs780094	GKCR	no
2	120548864	chr2:120548864:G:A	G	A	0.4285	439	-0.0035	0.0595	-0.9861	0.9532	rs17050272	INHBB	no
3	53066198	chr3:53066198:G:T	G	T	0.5672	439	0.0965	0.0584	28.3172	0.0984	rs6770152	SFMBT1	no
4	9914117	chr4:9914117:C:T	C	T	0.385	439	0.019672	0.058412	5.765425	0.736289	rs11722228	SLC2A9	no
4	9961141	chr4:9961141:T:C	T	C	0.1583	439	0.132849	0.079464	21.03837	0.094563	rs11942223	SLC2A9	no
4	9993558	chr4:9993558:G:C	G	C	0.8248	439	-0.21137	0.079101	-33.7809	0.007537	rs3775948	SLC2A9	no
4	9942428	chr4:9942428:A:G	A	G	0.1729	439	0.1557	0.0768	26.3829	0.0427	rs12498742	SLC2A9	yes
5	73135655	chr5:73135655:G:C	G	C	0.2832	439	0.0414	0.0705	8.3257	0.557	rs17632159	TMEM171	yes
6	25823216	chr6:25823216:A:T	A	T	0.552	439	0.081031	0.059006	23.27294	0.169674	rs1183201	SLC17A	no
6	7101851	chr6:7101851:T:C	T	C	0.6997	439	-0.0482	0.0625	-12.3396	0.4407	rs675209	RREB1	no
6	25821388	chr6:25821388:T:G	T	G	0.5542	439	0.0816	0.0588	23.6136	0.1652	rs1165151	SLC17A1	yes
6	43836834	chr6:43836834:T:G	T	G	0.7358	439	-0.0567	0.0677	-12.3548	0.4026	rs729761	VEGFA	no
7	73442719	chr7:73442719:A:G	A	G	0.1446	439	-0.2675	0.0793	-42.5252	0.0007	rs1178977	BAZ1B	no
7	151708919	chr7:151708919:C:T	C	T	0.2915	439	0.0502	0.062	13.074	0.4179	rs10480300	PRKAG2	no
8	23919493	chr8:23919493:A:G	A	G	0.4294	439	0.046	0.0618	12.0476	0.4566	rs17786744	STC1	no
8	75566533	chr8:75566533:C:T	C	T	0.4544	439	0.0525	0.0548	17.4563	0.3386	rs2941484	HNF4G	no
10	50886333	chr10:50886333:G:A	G	A	0.1893	439	0.059	0.0726	11.1934	0.4164	rs10821905	A1CF	yes
10	59709780	chr10:59709780:T:C	T	C	0.7745	439	0.0136	0.07	2.775	0.846	rs1171614	SLC16A9	no
11	64563990	chr11:64563990:A:G	A	G	0.4658	439	0.006287	0.057413	1.907401	0.912798	rs17300741	SLC22A11	no
11	64591337	chr11:64591337:G:A	G	A	0.6883	439	0.104108	0.063802	25.57521	0.102734	rs3825018	SLC22A12	yes
11	64778919	chr11:64778919:C:T	C	T	0.8701	439	0.082882	0.086458	11.08783	0.337743	rs606458	SF1	no
11	64566642	chr11:64566642:C:T	C	T	0.4658	439	0.0058	0.0574	1.7478	0.92	rs2078267	SLC22A11	no
11	64710591	chr11:64710591:G:A	G	A	0.8519	439	0.0643	0.0809	9.8295	0.4266	rs478607	NRXN2	no
12	51857488	chr12:51857488:G:T	G	T	0.3029	439	0.0139	0.0612	3.719	0.8198	rs7976059	ACVR1B	no
12	57450266	chr12:57450266:C:T	C	T	0.1891	439	0.1345	0.0763	23.1127	0.0778	rs3741414	INHBC	no
12	111569952	chr12:111569952:C:T	C	T	0.5011	439	-0.0264	0.0577	-7.9245	0.6473	rs653178	ATXN2	no
12	122141445	chr12:122141445:A:G	A	G	0.5046	439	0.0074	0.0577	2.2069	0.8986	rs7953704	B3GNT4	no
15	75866642	chr15:75866642:G:A	G	A	0.3916	439	-0.0083	0.0607	-2.2492	0.8913	rs1394125	UBE2Q2	no
15	98727906	chr15:98727906:A:G	A	G	0.6115	439	0.0263	0.0593	7.4873	0.6572	rs6598541	IGF1R	no
16	69529987	chr16:69529987:C:T	C	T	0.8565	439	-0.0737	0.0799	-11.5485	0.3563	rs7193778	NFAT5	no
16	79701090	chr16:79701090:G:A	G	A	0.3162	439	-0.0414	0.0651	-9.7654	0.525	rs7188445	MAF	yes
17	55287427	chr17:55287427:C:A	C	A	0.571	439	-0.1178	0.0585	-34.3828	0.0442	rs7224610	HLF	no

*Adjusted for age, sex, daily allopurinol dose, and PC1-5 (n=439 samples).

Gene information was retrieved with Annovar (version 2020-06-07). The rsID were retrieved with Ensembl REST API.

Abbreviations: CHR, chromosome; POS, position SE, standard error.
