# Supplementary material for: A Genome-Wide Association Study of Oxypurinol Concentrations in Patients Treated with Allopurinol
Source: J Pers Med. 2024 Jun 18;14(6):649. doi: 10.3390/jpm14060649 (PMC11204675; doi:10.3390/jpm14060649)
Supplement: Supplementary file 1 [file jpm-14-00649-s001.zip › S7 Table.pdf]

Table S7

GWAS results of genetic lookups for allopurinol-riboside concentrations (log) using a linear regression with ALLO-MHI biobank participants.

CHR	POS	rsID	Allele 1	Allele 2	Allele 2 frequency	N	BETA	SE	Tstat	p-value*	SNP	Gene	imputed
1	145711327	chr1:145711327:T:G	T	G	0.4636	439	-0.05385	0.061978	-14.0187	0.384932	rs1471633	PDZK1	no
1	240558252	chr1:240558252:C:T	C	T	0.315	439	-0.05073	0.069551	-10.4883	0.465717	rs1934341	GREM2	yes
1	155179017	chr1:155179017:C:T	C	T	0.3747	439	-0.0211	0.063	-5.3241	0.7372	rs11264341	TRIM46	no
2	27508073	chr2:27508073:T:C	T	C	0.5072	439	0.016674	0.060514	4.553202	0.782906	rs1260326	GKCR	no
2	27518370	chr2:27518370:T:C	T	C	0.5205	439	0.003723	0.060824	1.006294	0.951194	rs780094	GKCR	no
2	120548864	chr2:120548864:G:A	G	A	0.4285	439	0.0523	0.0625	13.3714	0.403	rs17050272	INHBB	no
3	53066198	chr3:53066198:G:T	G	T	0.5672	439	0.0475	0.0614	12.6032	0.4391	rs6770152	SFMBT1	no
4	9914117	chr4:9914117:C:T	C	T	0.385	439	-0.07652	0.061439	-20.2703	0.21299	rs11722228	SLC2A9	no
4	9961141	chr4:9961141:T:C	T	C	0.1583	439	0.147793	0.083582	21.15588	0.07702	rs11942223	SLC2A9	no
4	9993558	chr4:9993558:G:C	G	C	0.8248	439	-0.17934	0.0832	-25.9085	0.031116	rs3775948	SLC2A9	no
4	9942428	chr4:9942428:A:G	A	G	0.1729	439	0.1661	0.0808	25.4415	0.0398	rs12498742	SLC2A9	yes
5	73135655	chr5:73135655:G:C	G	C	0.2832	439	0.0962	0.0742	17.468	0.195	rs17632159	TMEM171	yes
6	25823216	chr6:25823216:A:T	A	T	0.552	439	0.001691	0.062064	0.438945	0.978266	rs1183201	SLC17A	no
6	7101851	chr6:7101851:T:C	T	C	0.6997	439	-0.0052	0.0657	-1.1936	0.9375	rs675209	RREB1	no
6	25821388	chr6:25821388:T:G	T	G	0.5542	439	0.0062	0.0618	1.6305	0.9197	rs1165151	SLC17A1	yes
6	43836834	chr6:43836834:T:G	T	G	0.7358	439	-0.0301	0.0713	-5.9353	0.6723	rs729761	VEGFA	no
7	73442719	chr7:73442719:A:G	A	G	0.1446	439	-0.1448	0.0834	-20.8137	0.0825	rs1178977	BAZ1B	no
7	151708919	chr7:151708919:C:T	C	T	0.2915	439	0.0683	0.0652	16.084	0.2946	rs10480300	PRKAG2	no
8	23919493	chr8:23919493:A:G	A	G	0.4294	439	-0.0053	0.065	-1.2541	0.935	rs17786744	STC1	no
8	75566533	chr8:75566533:C:T	C	T	0.4544	439	-0.013	0.0577	-3.9219	0.8211	rs2941484	HNF4G	no
10	50886333	chr10:50886333:G:A	G	A	0.1893	439	-0.0254	0.0764	-4.3575	0.7393	rs10821905	A1CF	yes
10	59709780	chr10:59709780:T:C	T	C	0.7745	439	-0.1157	0.0736	-21.3365	0.1161	rs1171614	SLC16A9	no
11	64563990	chr11:64563990:A:G	A	G	0.4658	439	0.027306	0.060388	7.487925	0.65114	rs17300741	SLC22A11	no
11	64591337	chr11:64591337:G:A	G	A	0.6883	439	0.169374	0.067107	37.61021	0.011605	rs3825018	SLC22A12	yes
11	64778919	chr11:64778919:C:T	C	T	0.8701	439	0.063826	0.090938	7.718063	0.482764	rs606458	SF1	no
11	64566642	chr11:64566642:C:T	C	T	0.4658	439	0.0315	0.0604	8.6411	0.6016	rs2078267	SLC22A11	no
11	64710591	chr11:64710591:G:A	G	A	0.8519	439	0.0876	0.0851	12.1006	0.3033	rs478607	NRXN2	no
12	51857488	chr12:51857488:G:T	G	T	0.3029	439	0.013	0.0644	3.1216	0.8406	rs7976059	ACVR1B	no
12	57450266	chr12:57450266:C:T	C	T	0.1891	439	0.01	0.0803	1.5588	0.9005	rs3741414	INHBC	no
12	111569952	chr12:111569952:C:T	C	T	0.5011	439	-0.0898	0.0607	-24.3484	0.1393	rs653178	ATXN2	no
12	122141445	chr12:122141445:A:G	A	G	0.5046	439	-0.0166	0.0607	-4.5129	0.784	rs7953704	B3GNT4	no
15	75866642	chr15:75866642:G:A	G	A	0.3916	439	0.0115	0.0639	2.8196	0.857	rs1394125	UBE2Q2	no
15	98727906	chr15:98727906:A:G	A	G	0.6115	439	-0.0255	0.0623	-6.5591	0.6826	rs6598541	IGF1R	no
16	69529987	chr16:69529987:C:T	C	T	0.8565	439	-0.0464	0.084	-6.5722	0.5809	rs7193778	NFAT5	no
16	79701090	chr16:79701090:G:A	G	A	0.3162	439	-0.0897	0.0685	-19.137	0.1901	rs7188445	MAF	yes
17	55287427	chr17:55287427:C:A	C	A	0.571	439	-0.0883	0.0616	-23.2835	0.1517	rs7224610	HLF	no

*Adjusted for age, sex, daily allopurinol dose, and PC1-5 (n=439 samples).

Gene information was retrieved with Annovar (version 2020-06-07). The rsID were retrieved with Ensembl REST API.

Abbreviations: CHR, chromosome; POS, position SE, standard error.
