# Supplementary material for: A Genome-Wide Association Study of Oxypurinol Concentrations in Patients Treated with Allopurinol
Source: J Pers Med. 2024 Jun 18;14(6):649. doi: 10.3390/jpm14060649 (PMC11204675; doi:10.3390/jpm14060649)
Supplement: Supplementary file 1 [file jpm-14-00649-s001.zip › S8 Table.pdf]

Table S8

GWAS results of genetic lookups for daily allopurinol dose (log) using a linear regression with ALLO-MHI biobank participants.

CHR	POS	rsID	Allele 1	Allele 2	Allele 2 frequency	N	BETA	SE	Tstat	p-value*	SNP	Gene	imputed
1	145711327	chr1:145711327:T:G	T	G	0.4636	439	0.007536	0.05882	2.178062	0.89806	rs1471633	PDZK1	no
1	240558252	chr1:240558252:C:T	C	T	0.315	439	-0.10443	0.06578	-24.1342	0.112391	rs1934341	GREM2	yes
1	155179017	chr1:155179017:C:T	C	T	0.3747	439	-0.0333	0.0598	-9.3201	0.5776	rs11264341	TRIM46	no
2	27508073	chr2:27508073:T:C	T	C	0.5072	439	0.018645	0.05743	5.65302	0.745445	rs1260326	GKCR	no
2	27518370	chr2:27518370:T:C	T	C	0.5205	439	-0.00388	0.05772	-1.1633	0.946466	rs780094	GKCR	no
2	120548864	chr2:120548864:G:A	G	A	0.4285	439	-0.0067	0.0593	-1.8919	0.9106	rs17050272	INHBB	no
3	53066198	chr3:53066198:G:T	G	T	0.5672	439	-0.0043	0.0583	-1.272	0.9409	rs6770152	SFMBT1	no
4	9914117	chr4:9914117:C:T	C	T	0.385	439	-0.06829	0.058236	-20.1345	0.240972	rs11722228	SLC2A9	no
4	9961141	chr4:9961141:T:C	T	C	0.1583	439	-0.05414	0.07928	-8.61437	0.49464	rs11942223	SLC2A9	no
4	9993558	chr4:9993558:G:C	G	C	0.8248	439	-0.01906	0.07894	-3.05837	0.809223	rs3775948	SLC2A9	no
4	9942428	chr4:9942428:A:G	A	G	0.1729	439	-0.0336	0.0767	-5.7187	0.6611	rs12498742	SLC2A9	yes
5	73135655	chr5:73135655:G:C	G	C	0.2832	439	-0.0611	0.0704	-12.3477	0.3849	rs17632159	TMEM171	yes
6	25823216	chr6:25823216:A:T	A	T	0.552	439	0.021003	0.058866	6.061035	0.721249	rs1183201	SLC17A	no
6	7101851	chr6:7101851:T:C	T	C	0.6997	439	0.0121	0.0624	3.1076	0.8463	rs675209	RREB1	no
6	25821388	chr6:25821388:T:G	T	G	0.5542	439	0.0257	0.0586	7.4843	0.6609	rs1165151	SLC17A1	yes
6	43836834	chr6:43836834:T:G	T	G	0.7358	439	0.1639	0.0671	36.354	0.0147	rs729761	VEGFA	no
7	73442719	chr7:73442719:A:G	A	G	0.1446	439	-0.0318	0.0791	-5.0707	0.6882	rs1178977	BAZ1B	no
7	151708919	chr7:151708919:C:T	C	T	0.2915	439	0.0293	0.0618	7.6698	0.6354	rs10480300	PRKAG2	no
8	23919493	chr8:23919493:A:G	A	G	0.4294	439	-0.1086	0.0615	-28.7319	0.0773	rs17786744	STC1	no
8	75566533	chr8:75566533:C:T	C	T	0.4544	439	0.064	0.0547	21.4014	0.242	rs2941484	HNF4G	no
10	50886333	chr10:50886333:G:A	G	A	0.1893	439	-0.049	0.0724	-9.3445	0.4985	rs10821905	A1CF	yes
10	59709780	chr10:59709780:T:C	T	C	0.7745	439	-0.0441	0.0699	-9.0347	0.5279	rs1171614	SLC16A9	no
11	64563990	chr11:64563990:A:G	A	G	0.4658	439	0.055112	0.057241	16.82018	0.335643	rs17300741	SLC22A11	no
11	64591337	chr11:64591337:G:A	G	A	0.6883	439	0.046783	0.063653	11.54624	0.462365	rs3825018	SLC22A12	yes
11	64778919	chr11:64778919:C:T	C	T	0.8701	439	0.145913	0.086093	19.68613	0.090107	rs606458	SF1	no
11	64566642	chr11:64566642:C:T	C	T	0.4658	439	0.0597	0.0573	18.213	0.297	rs2078267	SLC22A11	no
11	64710591	chr11:64710591:G:A	G	A	0.8519	439	-0.0047	0.0807	-0.7169	0.9538	rs478607	NRXN2	no
12	51857488	chr12:51857488:G:T	G	T	0.3029	439	0.1064	0.0609	28.7013	0.0805	rs7976059	ACVR1B	no
12	57450266	chr12:57450266:C:T	C	T	0.1891	439	-0.0622	0.0761	-10.7457	0.4138	rs3741414	INHBC	no
12	111569952	chr12:111569952:C:T	C	T	0.5011	439	-0.022	0.0576	-6.633	0.7023	rs653178	ATXN2	no
12	122141445	chr12:122141445:A:G	A	G	0.5046	439	0.0502	0.0576	15.1471	0.383	rs7953704	B3GNT4	no
15	75866642	chr15:75866642:G:A	G	A	0.3916	439	0.0427	0.0606	11.6372	0.4809	rs1394125	UBE2Q2	no
15	98727906	chr15:98727906:A:G	A	G	0.6115	439	0.0664	0.0591	19.0222	0.2612	rs6598541	IGF1R	no
16	69529987	chr16:69529987:C:T	C	T	0.8565	439	0.0335	0.0797	5.2784	0.6739	rs7193778	NFAT5	no
16	79701090	chr16:79701090:G:A	G	A	0.3162	439	-0.0643	0.0649	-15.2353	0.3224	rs7188445	MAF	yes
17	55287427	chr17:55287427:C:A	C	A	0.571	439	-0.0758	0.0583	-22.3064	0.1934	rs7224610	HLF	no

*Adjusted for age, sex, and PC1-5 (n=439 samples).

Gene information was retrieved with Annovar (version 2020-06-07). The rsID were retrieved with Ensembl REST API.

Abbreviations: CHR, chromosome; POS, position SE, standard error.
